# Supplementary material for: Differential Diagnosis of Alzheimer Disease vs. Mild Cognitive Impairment Based on Left Temporal Lateral Lobe Hypomethabolism on 18F-FDG PET/CT and Automated Classifiers
Source: Diagnostics (Basel). 2022 Oct 7;12(10):2425. doi: 10.3390/diagnostics12102425 (PMC9601187; doi:10.3390/diagnostics12102425)
Supplement: Supplementary file 1 [file diagnostics-12-02425-s001.zip › AD.pdf]

Brain 18F FDG PET/CT quantitative analysis on a 71-year-old female with AD.
MRI showed aspecific gliosis, MMSE was 26/30.

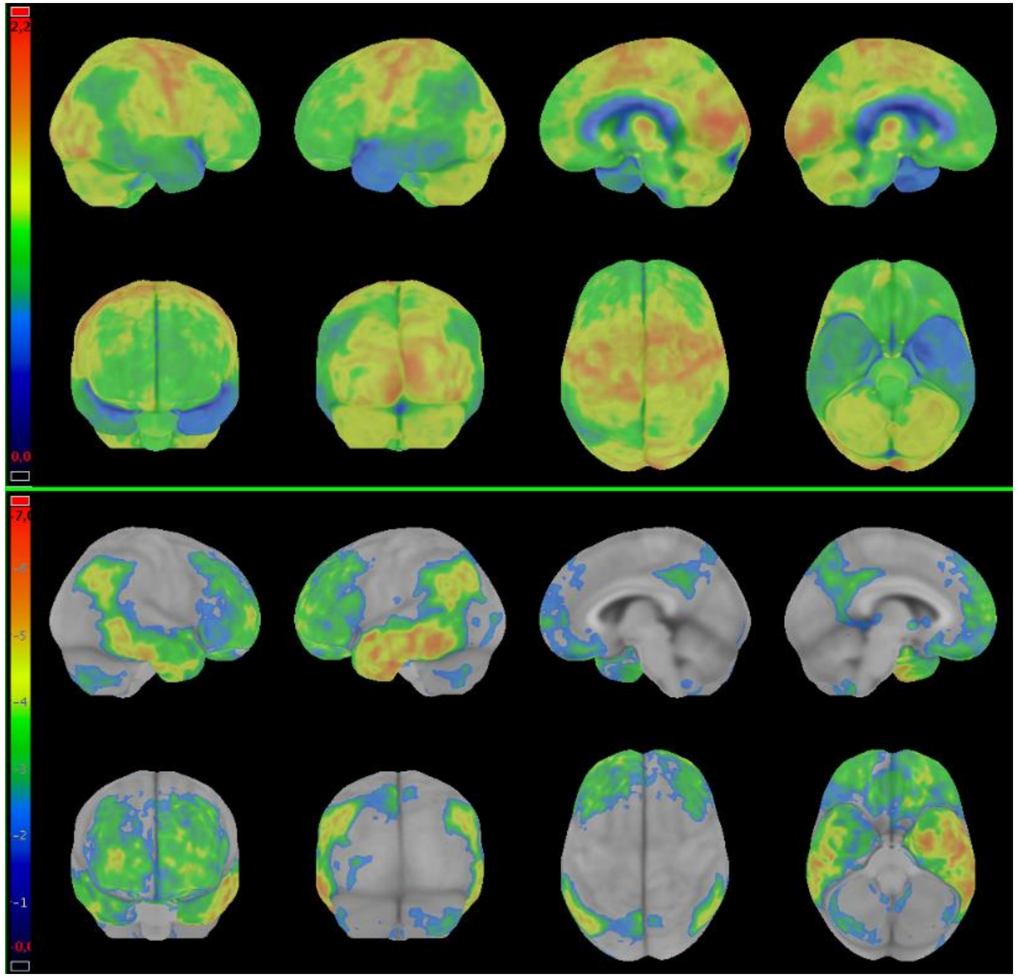

Cortical region	patient	normal	difference	Z score
Prefrontal Lateral R	1,19	1,51	-0,32	-2,88
Prefrontal Lateral L	1,12	1,49	-0,38	-3,45
Prefrontal Medial R	1,19	1,39	-0,20	-1,94
Prefrontal Medial L	1,13	1,40	-0,27	-2,53
Sensorimotor R	1,45	1,46	-0,01	-0,11
Sensorimotor L	1,42	1,47	-0,06	-0,48
Anterior Cingulate R	1,07	1,25	-0,18	-1,49
Anterior Cingulate L	1,01	1,25	-0,23	-1,99
Posterior Cingulate R	1,25	1,64	-0,38	-2,73
Posterior Cingulate L	1,20	1,63	-0,43	-3,10
Precuneus R	1,31	1,63	-0,32	-2,15
Precuneus L	1,17	1,61	-0,44	-3,17
Parietal Superior R	1,25	1,43	-0,18	-1,26
Parietal Superior L	1,23	1,40	-0,17	-1,32
Parietal Inferior R	1,15	1,47	-0,32	-2,71
Parietal Inferior L	1,04	1,45	-0,41	-3,40
Occipital Lateral R	1,38	1,54	-0,17	-1,29
Occipital Lateral L	1,27	1,53	-0,26	-2,03
Primary Visual R	1,49	1,68	-0,19	-1,18
Primary Visual L	1,51	1,67	-0,16	-1,01
Temporal Lateral R	0,98	1,33	-0,35	-3,71
Temporal Lateral L	0,85	1,32	-0,46	-4,92
Temporal Mesial R	0,83	1,04	-0,21	-3,10
Temporal Mesial L	0,72	1,04	-0,31	-4,59
Cerebellum Whole	1,14	1,24	-0,10	-1,49
Pons	1,00	1,00	-0,00	0,00
