# Supplementary material for: Differential Diagnosis of Alzheimer Disease vs. Mild Cognitive Impairment Based on Left Temporal Lateral Lobe Hypomethabolism on 18F-FDG PET/CT and Automated Classifiers
Source: Diagnostics (Basel). 2022 Oct 7;12(10):2425. doi: 10.3390/diagnostics12102425 (PMC9601187; doi:10.3390/diagnostics12102425)
Supplement: Supplementary file 1 [file diagnostics-12-02425-s001.zip › MCI.pdf]

Brain 18F FDG PET/CT quantitative analysis on a 70-year-old male with MCI.
MRI showed no abnormalities, MMSE was 30/30.

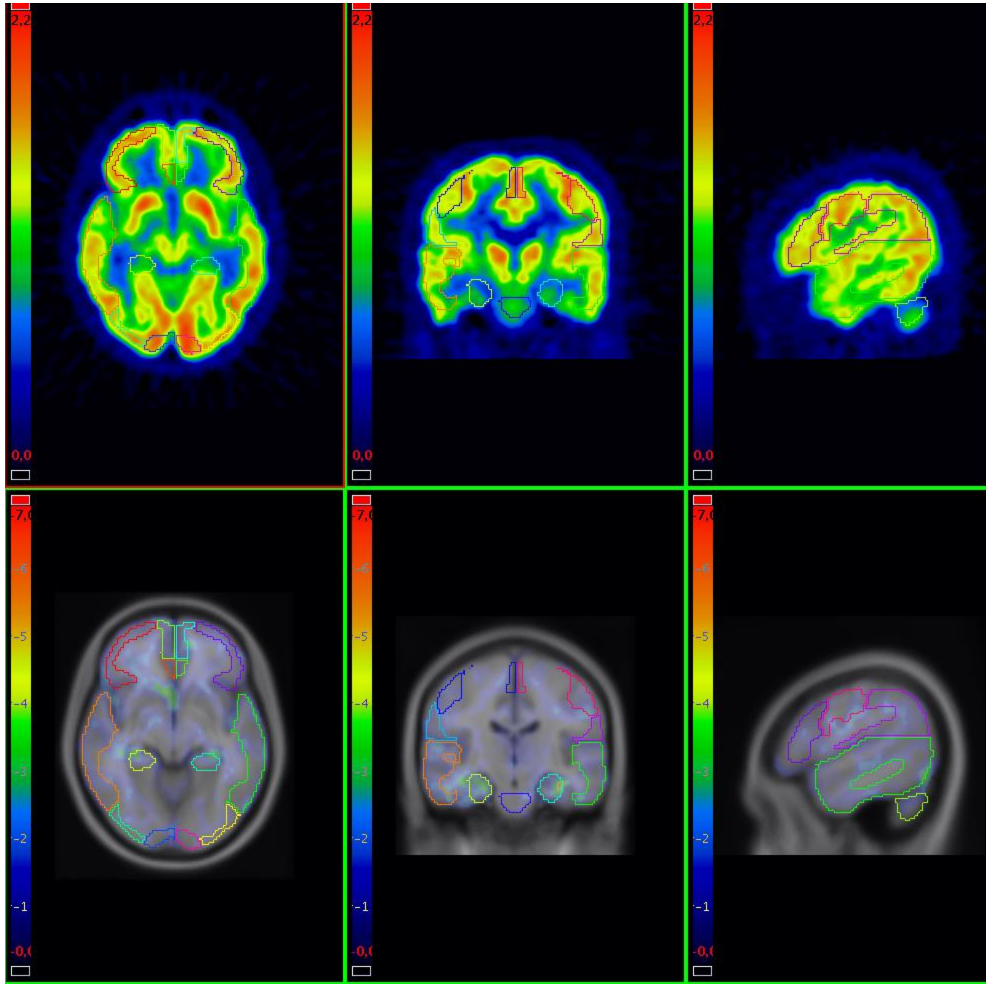

Cortical region	patient	normal	difference	Z score
Prefrontal Lateral R	1,47	1,58	−0,11	−0,80
Prefrontal Lateral L	1,46	1,57	−0,12	−0,80
Prefrontal Medial R	1,34	1,45	−0,11	−0,83
Prefrontal Medial L	1,33	1,46	−0,13	−0,93
Sensorimotor R	1,45	1,51	−0,05	−0,34
Sensorimotor L	1,47	1,51	−0,04	−0,28
Anterior Cingulate R	1,15	1,32	−0,17	−1,16
Anterior Cingulate L	1,16	1,30	−0,14	−1,04
Posterior Cingulate R	1,36	1,68	−0,32	−1,68
Posterior Cingulate L	1,37	1,67	−0,30	−1,54
Precuneus R	1,53	1,68	−0,15	−0,83
Precuneus L	1,53	1,66	−0,12	−0,67
Parietal Superior R	1,46	1,48	−0,02	−0,11
Parietal Superior L	1,41	1,45	−0,04	−0,22
Parietal Inferior R	1,42	1,54	−0,12	−0,79
Parietal Inferior L	1,45	1,53	−0,08	−0,49
Occipital Lateral R	1,47	1,60	−0,13	−0,81
Occipital Lateral L	1,52	1,58	−0,07	−0,39
Primary Visual R	1,62	1,74	−0,12	−0,64
Primary Visual L	1,65	1,73	−0,07	−0,38
Temporal Lateral R	1,22	1,39	−0,18	−1,47
Temporal Lateral L	1,28	1,39	−0,11	−0,84
Temporal Mesial R	0,80	1,06	−0,26	−3,34
Temporal Mesial L	0,90	1,06	−0,16	−2,05
Cerebellum Whole	1,24	1,25	−0,02	−0,25
Pons	1,00	1,00	−0,00	0,00
